# Is There a Relationship Between Movement and Sleep Disturbances in Essential Tremor?

**DOI:** 10.3390/brainsci16050504

**Published:** 2026-05-07

**Authors:** Giulia Paparella, Adriana Martini, Anna Sofia Grandolfo, Matteo Panfili, Luca Angelini, Martina De Riggi, Simone Aloisio, Daniele Birreci, Annalisa Maraone, Francesco Saverio Bersani, Matteo Bologna

**Affiliations:** 1 IRCCS Neuromed, 86077 Pozzilli (IS), Italy; giulia.paparella1@uniba.it (G.P.); luca.angelini@uniroma1.it (L.A.); 2 Neurophysiopathology Unit, Department of Translational Biomedicine and Neuroscience (DiBraiN), University of Bari Aldo Moro, 70124 Bari, Italy; 3 Department of Human Neurosciences, Sapienza University of Rome, 00185 Rome, Italy; adriana.martini@uniroma1.it (A.M.); sofia.grandolfo01@gmail.com (A.S.G.); matteo.panfili@uniroma1.it (M.P.); martina.deriggi@uniroma1.it (M.D.R.); simone.aloisio@uniroma1.it (S.A.); daniele.birreci@uniroma1.it (D.B.); annalisa.maraone@uniroma1.it (A.M.); 4 Department of Translational and Precision Medicine, Sapienza University of Rome, 00185 Rome, Italy; 5 Department of Medico-Surgical Sciences and Biotechnologies, Sapienza University of Rome, 04100 Rome, Italy; francescosaverio.bersani@uniroma1.it

**Keywords:** essential tremor, bradykinesia, sleep disorders, kinematic analysis

## Abstract

**Highlights:**

**What are the main findings?**

- Essential tremor (ET) is a heterogeneous disorder with motor and non-motor features, possibly due to Lewy body pathology
- No significant association was found between finger-tapping kinematics and insomnia severity in ET patients

**What are the implications of the main findings?**

- Movement and sleep disturbances in ET may reflect distinct pathophysiological mechanisms (cerebellar–basal ganglia vs. brainstem involvement)
- Larger multimodal studies are needed to better clarify the pathophysiological consequences of Lewy body pathology in ET

**Abstract:**

Background: Essential tremor (ET) is a clinically heterogeneous disorder involving motor and non-motor features. Voluntary movement abnormalities, including movement slowness (bradykinesia), have been frequently described in ET. Among non-motor symptoms, insomnia is also frequently reported, raising the possibility of shared underlying mechanisms with bradykinesia (e.g., brainstem noradrenergic dysfunction involving the locus coeruleus due to Lewy body pathology). We investigated the relationship between movement abnormalities, as objectively quantified using finger-tapping kinematic analysis, and sleep disturbances in ET. Methods: A subsample of 29 ET patients included in a previous study underwent kinematic analysis of finger-tapping. Insomnia was evaluated using the Insomnia Severity Index (ISI). Patients were stratified according to the presence of bradykinesia (e.g, movement slowness during finger-tapping) and insomnia (ISI ≥ 8). Group comparisons and correlational analyses were performed to assess the association between kinematic measures of bradykinesia, insomnia severity and other clinical features. Results: Fourteen subjects (48.3%) exhibited bradykinesia on kinematic analysis, and eleven patients (37.9%) met the criteria for insomnia. The prevalence of insomnia was similar between patients with and without bradykinesia. Likewise, no significant differences in finger-tapping kinematics were observed between ET patients with and without insomnia. Kinematic measures of bradykinesia did not correlate with ISI scores (all *p* > 0.05), whereas ISI scores were significantly positively correlated with clinical tremor severity. Conclusions: The current kinematic analysis suggests no relationship between movement abnormalities and sleep disturbances in ET. While bradykinesia in ET possibly relies on the dysfunction of cerebellar–basal ganglia circuits, insomnia in ET may reflect prominent brainstem dysfunction. Larger studies integrating kinematic assessments, neuroimaging, and longitudinal designs are needed to clarify the relationship between movement and sleep disturbances in ET

## 1. Introduction

Essential tremor (ET) is one of the most common movement disorders worldwide and is traditionally defined by the presence of bilateral upper limb action tremor [1,2]. However, growing evidence has progressively challenged this purely motor-centered definition, highlighting ET as a clinically heterogeneous disorder characterized by both motor and non-motor manifestations [2,3,4,5,6]. In addition to action tremor, patients frequently exhibit subtle additional motor signs, including impaired execution of voluntary movement [7,8,9,10,11,12,13], as well as a wide spectrum of non-motor symptoms, including cognitive impairment and neuropsychiatric disturbances [5,7,9,14,15,16,17,18,19,20,21,22]. The recognition of ET’s complexity has raised interest in identifying clinical subtypes and pathophysiological mechanisms that may underlie its marked phenotypic variability [1,6,23,24].

Among the additional motor features described in ET, several clinical and kinematic studies have shown that patients with ET may exhibit mild slowing of movement execution (i.e., bradykinesia) compared to healthy subjects, independently of tremor severity [8,9,12,13,25,26]. The pathophysiologic basis of this phenomenon remains unclear. Some studies suggest that subtle dopaminergic dysfunction at the level of the basal ganglia may contribute to motor slowing [21,27,28,29]. Other mechanisms may involve alterations within cerebellar and cortical circuits [5,13,30,31].

Alongside motor manifestations, sleep disturbances are increasingly recognized as relevant non-motor features of ET [5,32,33,34]. Insomnia appears particularly common, with many patients reporting difficulties initiating or maintaining sleep, early awakenings, or non-restorative sleep. Although sleep disorders have been extensively investigated in PD and other synucleinopathies, their clinical significance in ET remains less clearly defined [5,32,33,34]. Given that ET is a clinically heterogeneous condition, sleep-related manifestations may vary depending on several factors, including age at onset, disease duration, tremor distribution and severity, cognitive status, and the presence of other non-motor symptoms. However, previous studies have primarily focused on the prevalence of sleep disturbances in ET, whereas their relationship with other motor and non-motor features has received less attention. For example, no previous study has investigated a possible link between movement and sleep disturbances in ET. This potential link may involve common pathophysiological mechanisms; e.g., the involvement of locus coeruleus (LC), a brainstem nucleus that plays a central role in both motor regulation and sleep–wake control. Through its widespread noradrenergic projections, the LC modulates cortical excitability, cerebellar output, and basal ganglia activity, all of which are critical for normal movement execution [35,36,37]. At the same time, the LC is a key regulator of arousal, attention, and sleep–wake transitions [38,39,40,41]. Neuropathological studies have recently demonstrated the presence of Lewy pathology within the LC in a subset of patients with ET [42,43,44,45], suggesting that noradrenergic degeneration may contribute to both motor and non-motor manifestations. In this framework, LC dysfunction could plausibly account for impaired movement execution through altered modulation of motor circuits.

Based on these considerations, the present study aimed to specifically investigate the potential relationship between movement and sleep disturbances in ET. We hypothesized that patients with slowed movement execution would more frequently present insomnia, possibly reflecting shared underlying mechanisms related to LC dysfunction and the noradrenergic system. By examining the association between movement and sleep disturbances, this study seeks to contribute to a more integrated understanding of ET pathophysiology and clinical heterogeneity.

## 2. Materials and Methods

### 2.1. Participants

A subsample of 29 subjects from our previous report [5] also underwent recording of repetitive finger-tapping kinematics, and thus these participants were included in the present study. Patients were diagnosed with ET according to the current consensus criteria [1]. Participants were recruited from the movement disorder outpatient clinic of the Policlinico Umberto I, Department of Human Neurosciences, Sapienza University of Rome, Italy. Inclusion and exclusion criteria are detailed in our previous paper [5]. Patients receiving anti-tremor medications were evaluated after treatment withdrawal [7,8,9]. Specifically, medications were discontinued 24 h before evaluation for propranolol and benzodiazepines, and 48 h prior for primidone and topiramate [7,8,9]. All participants provided written informed consent prior to inclusion. The study was approved by the local institutional review board and conducted in accordance with the ethical standards of the Declaration of Helsinki.

### 2.2. Clinical Evaluation

Key demographic and clinical information was collected from all participants, as detailed in [5]. All participants underwent a detailed neurological examination. Tremor severity was assessed using the Essential Tremor Rating Assessment Scale (TETRAS) [46], which includes the performance subscale (TETRAS_P_) and the activities of daily living subscale (TETRAS_ADL_), with upper limb tremor scores and the number of body parts affected by tremor derived as reported in [5]. All possible soft signs (e.g., subtle bradykinesia, questionable dystonic postures and impaired tandem gait) were systematically evaluated following the same protocol adopted in [5], including clinical scales such as the Movement Disorder Society Unified Parkinson’s Disease Rating Scale (MDS-UPDRS) Part III and the Scale for the Assessment and Rating of Ataxia (SARA). Sleep disturbances were evaluated using the Insomnia Severity Index (ISI) [47], with scores ≥ 8 indicating insomnia. Participants were also asked about vivid dreams or abnormal nocturnal movements suggestive of restless legs syndrome or of REM sleep behavior disorder. Perceived stress levels were measured with the Perceived Stress Scale (PSS) [48]. Global cognitive function was screened using the Montreal Cognitive Assessment (MoCA) [49]. We also adopted the Hospital Anxiety and Depression Scale (HADS) to score anxiety and depressive symptoms [50]. Finally, participants provided self-reported measures of overall quality of life (QoL) and health-related quality of life (HR-QoL).

### 2.3. Kinematic Recordings and Analyses

Kinematics of repetitive finger-tapping were recorded using a 3-D optoelectronic system (SMART motion system, BTS, Milan, Italy), consisting of three infrared cameras that detected the movement in three dimensional space of reflective markers placed on the body segments to be analyzed [7,9,12,51]. Reflective markers were positioned as follows: two markers were placed on the distal phalanges of the first and second fingers, and the remaining four markers were placed on the head and base of the second metacarpal, the base of the fifth metacarpal, and the wrist, to establish a reference plane on the hand and exclude any contamination of finger movements due to undesired hand movements. The cameras had a sampling rate of 120 Hz [7,9,12,51]. Motion analysis was conducted offline using dedicated software (SMART Analyzer 1.10.0470, BTS Engineering, Milan, Italy), to calculate the kinetic variables of interest, including the number of movements, amplitude and velocity of the movement expressed in degrees and degrees per second. Additional parameters included the decrement in amplitude and velocity during repetitive movements (i.e., the sequence effect) expressed as degree/n mov and (degree/sec)/n mov, respectively, and the coefficient of variation (CV), an expression of rhythm defined as the ratio of standard deviation (SD) to the mean value of intervals in three finger-tapping, with higher values indicating less rhythmic movement [7,9,12,51].

To provide a comprehensive kinematic assessment, we also evaluated tremor [9,10]. Upper limb postural tremor was recorded during two postures with the arms outstretched in front of the chest (posture 1—P1) and with the arms flexed at the elbows (i.e., lateral “wing beating” posture; posture 2—P2) during three 45 s recordings per position [9,10]. Upper limb rest tremor was measured while patients were seated with the upper limbs completely relaxed and resting comfortably on a plane in front of them. Kinetic tremor was recorded during three 15 s recordings while patients performed a pointing task. Tremor analysis was performed using dedicated software (SMART Analyzer, BTS Engineering, Italy). The root mean square (RMS) of the acceleration traces of the reference markers in 3D space was measured to determine the magnitude of postural and rest tremor of the upper limbs and head tremor, expressed in GRMS^2. Power spectra were calculated by means of fast Fourier transformation. We then measured the dominant frequency peak (Hz) of tremor. For kinetic tremor, we used an algorithm that determined the curvature index—CI (i.e., arm endpoint average path length/length of a straight line joining the initial and final positions), considered as an index of movement homogeneity [9,10].

### 2.4. Statistical Analysis

Data distribution was visually inspected and formally assessed using the Shapiro–Wilk test. As several variables showed non-normal distributions, non-parametric statistical tests were applied. Participants were stratified according to (i) the presence of subtle bradykinesia, kinematically assessed, into ET non-slow (ET-ns) and ET slow (ET-s) groups based on movement velocity using a median split procedure, in line with previous kinematic studies employing the same methodological framework [31,52], and (ii) the presence of insomnia in patients without (ET-ni) and with insomnia (ET-i) based on ISI ≥ 8 [5]. Categorical variables—including sex, the presence of tremor in the head, face, voice, lower limbs, rest tremor, as well as clinical soft signs such as questionable bradykinesia, dystonia, and impaired tandem gait—were expressed as frequencies and compared between groups using Fisher’s exact test. Between-group differences in continuous clinical variables (e.g., tremor severity scores, upper limb action and rest tremor scores, SARA, MoCA, quality-of-life measures, as well as kinematic features of finger-tapping and tremor features) were assessed using the Mann–Whitney U test. Spearman’s rank correlation analysis was performed across the entire cohort to explore associations between insomnia severity, demographic variables (e.g., age, age at tremor onset), clinical features (TETRAS_TOT_, TETRAS_P_, TETRAS_ADL_, upper limbs action and rest tremor severity, SARA, MDS-UPDRS items 3.4–3.8, MoCA, HADS, PSS, and BRS scores), and kinematics. To further assess the robustness of the correlation findings and to account for potential confounding factors, linear regression models were performed with movement velocity as the dependent variable and ISI as the main predictor. Age and TETRAS scores were included as covariates. Standardized β coefficients, standard errors, t-values, and *p*-values are reported. Model fit was assessed using R^2^ (and adjusted R^2^). Results are reported as mean ± standard deviation. Statistical significance was set at *p* < 0.05, with multiple comparisons corrected using the false discovery rate (FDR); i.e., *p_adj* (FDR) [53]. Effect size estimates were also calculated. Specifically, we used rank-biserial correlation as an effect size measure for Mann–Whitney U tests, and appropriate measures of association (e.g., φ coefficient) were used for categorical variables. For the main self-reported clinical scale (i.e., ISI), we tested the internal consistency using the Cronbach’s alpha, with values > 0.7 indicating a good internal consistency. Data analysis was carried out using STATISTICA^®^ Version 6 (TIBCO Software Inc., Palo Alto, CA, USA) and implemented using Jamovi (version 2.6).

## 3. Results

### 3.1. General Results

The sample included in the present study consisted of 29 participants, 15 of whom were female (51.7%). The mean age was 66.6 ± 14.7 years, the mean age at tremor onset 47.1 ± 20.8 years. Sixteen patients (55.2%) reported a family history of tremor. Twenty-three individuals (79.3%) reported caffeine use, while 20 (38.9%) reported current or past cigarette smoking. Comorbidities are listed in Table S1. The mean years of education were 9.25 ± 5.13. Concerning employment status, 18 subjects (62.1%) reported being unemployed or retired due to tremor-related disability. Twelve patients (41.4%) used propranolol alone or in combination with other drugs, 6 patients (20.7%) used benzodiazepines, 8 (27.6%) used gabapentinoids, and 2 (6.9%) used antiepileptics.

All patients had bilateral upper limb action tremor; additionally, 11 (37.9%) had head tremor, 5 (17.2%) face tremor, 13 (44.8%) voice tremor, and 10 (34.5%) lower limb tremor. Rest tremor was observed in 19 patients (65.5%), and questionable bradykinesia in 10 (34.5%), though none met full criteria for parkinsonism [1,8,9,10]. One patient (3.4%) showed questionable dystonic posturing, and 10 (34.5%) exhibited mild impaired tandem gait. The mean TETRAS_TOT_ was 36.3 ± 16.2 (TETRAS_P_: 22.4 ± 9.54; TETRAS_ADL_: 13.9 ± 8.25). The mean Movement Disorder Society Unified Parkinson’s Disease Rating Scale (MDS-UPDRS) Part III score in the overall patient sample was 10.8 ± 7.85. The mean score of the Scale for the Assessment and Rating of Ataxia (SARA) was 2.05 ± 1.92. The mean ISI score was 7 ± 5.25 (Cronbach’s alpha 0.85). Six (20.7%) reported vivid dreams. The mean PSS score was 14.8 ± 5.69. The average MoCA score was 25.8, with 11 patients scoring below 26. Mean HADS scores were 6.03 ± 3.30 for anxiety and 5.10 ± 3.62 for depression. QoL and HR-QoL were 75.5 ± 17.3 and 70 ± 25.5, respectively.

Concerning kinematic finger-tapping assessment, the mean number of tapping movements was 41.4 ± 10.1. The mean velocity and amplitude were 1020 ± 266 degrees/s and 47.9 ± 10.8 degrees, respectively. Overall tapping rhythm, expressed as the CV, averaged 0.09 ± 0.04. Mean amplitude decrement over the trial was −0.14 ± 0.23 degrees/n mov, while the decrement in tapping velocity was −5.63 ± 4.15 (degrees/sec)/n mov. Regarding tremor kinematics, the mean rest tremor frequency was 5.83 ± 1.02 Hz, with a mean amplitude of 0.039 ± 0.061 GRMS^2. During postural conditions, the mean tremor frequency was 5.18 ± 0.92 Hz in P1 and 5.86 ± 1.54 Hz in P2, with corresponding amplitudes of 0.073 ± 0.049 GRMS^2 and 0.273 ± 0.407 GRMS^2, respectively. The mean CI value, which assesses kinetic tremor, was 1.23 ± 0.88.

### 3.2. Subgroup Analysis

#### 3.2.1. ET-s Versus ET-ns

**Figure 1:**
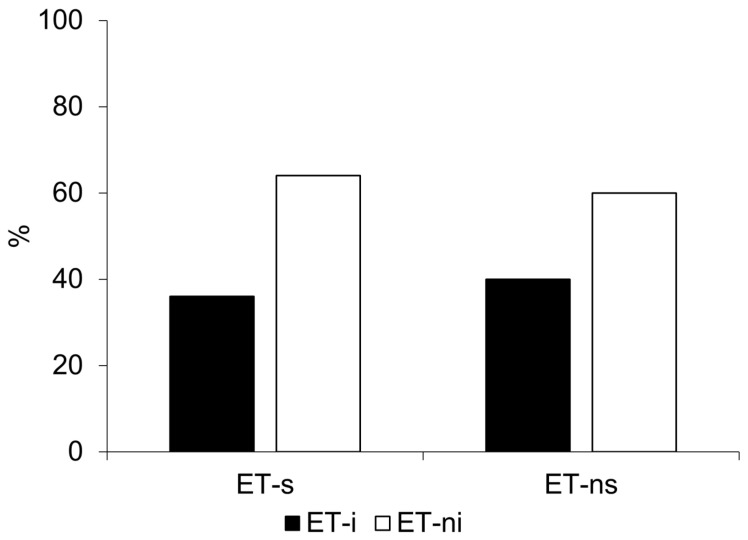
Percentage of patients with essential tremor (ET) stratified by the presence of subtle bradykinesia, kinematically assessed based on movement velocity using a median split procedure, and by the presence of insomnia. Patients were categorized as ET non-slow (ET-ns) or ET slow (ET-s) and further subdivided into those without insomnia (ET-ni) and with insomnia (ET-i), defined by the Insomnia Severity Index (ISI) ≥ 8.

Fourteen subjects (48.3%) were included in the ET-s subgroup, while 15 patients (51.7%) were in the ET-ns subgroup. Although ET-s patients were older than ET-ns, this difference did not survive correction for multiple comparisons (*p* = 0.006, *p_adj* (FDR) = 0.05, rank-biserial correlation = −0.595, Table 1). No significant differences were observed between subgroup in the main demographic and clinical characteristics, including age at tremor onset, sex distribution, family history of tremor, or tremor distribution (Table 1 and Table 2). Upper limb tremor severity showed a trend toward higher action tremor in ET-ns (*p* = 0.046, rank-biserial correlation = 0.428), but this difference did not survive correction for multiple comparisons (*p_adj* (FDR) = 0.098). As expected, MDS-UPDRS Part III scores were significantly higher in ET-s patients compared with ET-ns (15.4 ± 8.39 vs. 6.47 ± 4.10, *p* = 0.001, *p_adj* (FDR) = 0.016, rank-biserial correlation = −0.709). Cognitive disturbances were slightly more frequent in ET-ns (60.0% vs. 14.3%, *p* = 0.011, φ value = 0.471), and MoCA scores showed a trend toward being lower in ET-ns (24.8 ± 2.57 vs. 26.9 ± 1.27, *p* = 0.013); however, these differences did not survive correction for multiple comparisons (*p_adj* (FDR) = 0.154 and 0.052, respectively). Importantly, the occurrence of insomnia (ISI ≥8) was similar between groups (40.0% vs. 35.7%, *p* = 0.812, Figure 1), and mean ISI scores were comparable between subgroups.

Finally, regarding tremor kinematics, no significant differences were observed between groups for rest tremor amplitude, postural tremor frequency and amplitude (P1 and P2), or kinetic tremor measures (Table 1). Rest tremor frequency tended to be higher in ET-ns compared with ET-s (6.33 ± 0.73 vs. 5.48 ± 1.07, *p* = 0.034, rank-biserial correlation = −0.614), but this difference, again, did not survive correction for multiple comparisons (*p_adj* (FDR) = 0.119).

#### 3.2.2. ET-i Versus ET-ni

Eleven out of 29 patients (37.9%) reported insomnia (ET-I, ISI ≥ 8) (Table 3 and Table 4), and the analysis showed no group differences in sex distribution and age. Similarly to what we observed in our previous report on these participants in the context of a larger sample [7], clinical variables such as age at tremor onset, positive family history of tremor, and tremor distribution did not differ between groups. However, significant group differences emerged for clinical measures of tremor severity. ET-i patients showed higher TETRAS_TOT_ (*p* = 0.002, *p_adj* (FDR) = 0.016, rank-biserial correlation = 0.687), as well as higher TETRAS_ADL_ (*p* = 0.006, *p_adj* (FDR) = 0.024, rank-biserial correlation = 0.606), TETRAS_P_ (*p* = 0.004, *p_adj* (FDR) = 0.02, rank-biserial correlation = 0.611) (Table 3). Upper limb tremor severity was greater in the ET-i subgroup, both for action tremor (*p* = 0.013, *p_adj* (FDR) = 0.039) and rest tremor (*p* = 0.035), although the latter difference did not survive FDR correction (*p_adj* (FDR) = 0.079).

No significant differences were observed between subgroups for rest tremor, subtle bradykinesia, questionable dystonia, impaired tandem gait, or cognitive disturbances (Table 4). No significant differences were observed for MDS-UPDRS Part III or SARA scores (Table 3). Overall MoCA scores slightly differed between subgroups, being lower in ET-i (*p* = 0.025, rank-biserial correlation = −0.454), although this difference did not survive correction for multiple comparisons (*p_adj* (FDR) = 0.054). Vivid dreams were reported in a similar percentage of patients. Stress levels were slightly lower in the ET-i subgroup (*p* = 0.018, *p_adj* (FDR) = 0.045, rank-biserial correlation = −0.555). HADS, QoL and HR-QoL did not differ between subgroups (Table 3).

**Figure 2:**
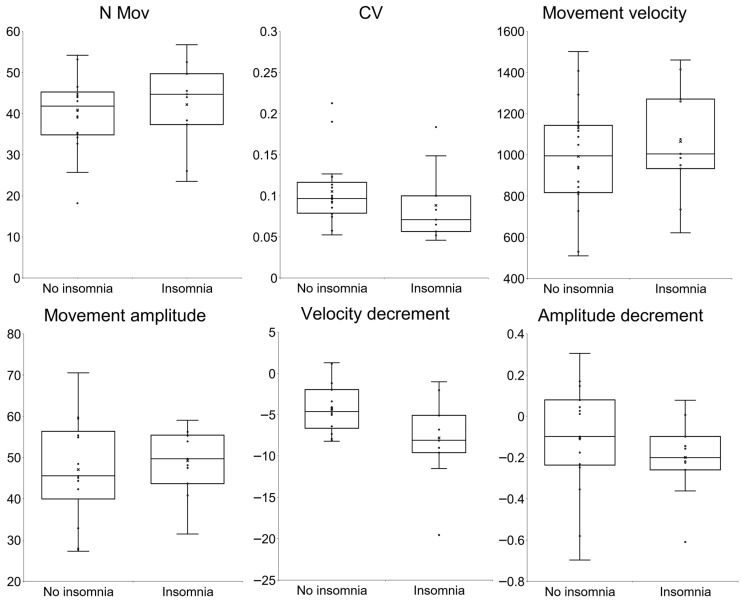
Kinematic variables of repetitive finger-tapping in essential tremor patients divided into subjects with and without insomnia according to the Insomnia Severity Index. N mov: number of movements, CV: coefficient of variation. Movement velocity is expressed in degrees/sec, movement amplitude in degrees. Movement velocity and amplitude decrement are expressed in (degrees/sec)/n mov and (degrees/n mov), respectively. The box represents the interquartile range. The horizontal line inside the box indicates the median value of the distribution. The whiskers extend to the minimum and maximum values within the non-outlier range. Dots represent individual values. The cross (×) indicates the mean value. Data were compared by Mann–Whitney U test.

The kinematic analysis revealed no significant differences in the mean number of movements (*p* = 0.739), movement velocity and amplitude (*p* = 0.489, and *p* = 0.653), CV (*p* = 0.616), and amplitude decrement (*p* = 0.282) (Table 3 and Figure 2). Consistently, the number of patients with subtle bradykinesia as kinematically assessed was similar in the ET-i and ET-ni subgroups (Table 4 and Figure 1). A significant difference was observed in the mean decrement of velocity over the trial, which was greater in the ET-i subgroup (*p* = 0.021, rank-biserial correlation = −0.515) (Table 3 and Figure 2). Nevertheless, this difference did not remain significant after FDR correction for multiple comparisons (*p_adj* (FDR) = 0.109). Finally, no significant differences were observed between groups for tremor kinematic parameters (Table 3).

### 3.3. Correlation Analysis

Consistently with our previous findings on these participants in the context of a larger sample [7], Spearman’s correlation analyses showed that ISI was positively associated with several clinical measures of tremor severity. Specifically, ISI showed significant positive correlations with TETRAS_TOT_ (ρ = 0.581, *p* < 0.001), TETRAS_ADL_ (ρ = 0.525, *p* = 0.003), TETRAS_P_ (ρ = 0.538, *p* = 0.003), and action upper-limb severity (ρ = 0.535, *p* = 0.003). Additionally, there was a trend toward a positive correlation with rest upper-limb severity (ρ = 0.328, *p* = 0.083), though this did not reach statistical significance. ISI, however, did not correlate with finger-tapping performance or tremor kinematics (all *p* > 0.05, Table 5).

### 3.4. Linear Regression Analysis

The overall model explained 13% of the variance in movement velocity (R^2^ = 0.130). In the adjusted analysis, ISI was not significantly associated with movement velocity (β = −9.96, SE = 12.38, t = −0.805, *p* = 0.429). Neither age nor TETRAS total score emerged as significant predictors of movement velocity.

## 4. Discussion

This study investigated the relationship between subtle bradykinesia and insomnia in patients with ET. Based on previous evidence suggesting a possible interaction between motor control and sleep regulation within the LC [35,36,37,39,41], which has been implicated in the pathophysiology of ET [5,44,45], we hypothesized that bradykinetic features might be associated with insomnia in this population. To explore this potential relationship, we adopted a comprehensive approach combining detailed clinical assessment, objective kinematic analysis of finger-tapping movements, and standardized evaluation of insomnia severity. Our findings contribute to clarifying the pathophysiological mechanisms underlying the so-called ‘soft signs’ observed in ET and provide further insight into the clinical heterogeneity of the disorder [1,2].

Consistent with previous reports, a substantial proportion of the patients in our cohort exhibited subtle bradykinesia on clinical examination [3,9,10,13,29,54]. Kinematic analysis provided objective information on movement parameters, specifically movement velocity, amplitude, rhythm, and velocity/amplitude decrement during movement execution, thereby enabling a more precise characterization of the bradykinesia complex [55] in ET. These findings further support movement slowness as the most prominent motor abnormality in affected patients [12,13]. Likewise, insomnia was reported by a considerable number of patients, confirming that sleep disturbances represent a relevant non-motor manifestation of ET and contribute to disease-related burden [5,32,33]. However, contrary to our initial hypothesis, we did not observe a higher occurrence of insomnia in patients with subtle bradykinesia, nor significant differences in bradykinesia features between patients with and without insomnia. Again, we did not find any significant correlations between kinematic features and insomnia severity. Importantly, the lack of association between bradykinesia and insomnia was consistent across different analytical approaches, including regression analysis controlling for age and tremor severity. These findings suggest that, although both symptoms are relatively common in ET, they may arise from partially independent pathophysiological mechanisms.

The neurobiological mechanisms underlying sleep disturbances in ET remain incompletely understood. Several brain regions and neurotransmitter systems are involved in sleep regulation, among which the LC plays a particularly important role [38,39,40,41,44]. Through its widespread noradrenergic projections, the LC contributes to the regulation of arousal, attention, and sleep–wake transitions. Neuropathological studies have reported Lewy pathology in a subset of ET cases, with the LC representing one of the most frequently involved brainstem structures [42,43,44,45,56]. Accordingly, recent neuroimaging studies found a lower contrast-to-noise ratio of LC in MRI scans in ET compared to controls [45,57]. Interestingly, similar pathological changes occur in PD, where sleep disturbances are considered part of the prodromal phase and have been linked to early brainstem degeneration. However, the relationship between structural changes in the LC and their functional implications in ET is still unclear. Recent longitudinal evidence has further explored this issue [58]. In a postmortem study, sleep characteristics collected prospectively over several years were associated with neuropathological findings. Lewy pathology was detected in more than 20% of cases, frequently involving the LC, and longer sleep latency was associated with the presence of Lewy pathology. These observations support the hypothesis that brainstem degeneration, particularly noradrenergic or sleep-regulating networks, may contribute to sleep dysfunction in ET patients [58]. This hypothesis is further reinforced by a prospective population-based study in which daily sleep duration was assessed in individuals over 65 years of age at baseline and again three years later [59]. The authors reported a higher risk of incident ET in short sleepers compared with long sleepers, suggesting that reduced daily sleep duration may represent a premotor marker of ET [59]. Nevertheless, mechanisms beyond LC dysfunction may be involved. Alterations in other neurotransmitter systems, including GABAergic and glutamatergic pathways, particularly within thalamic circuits, may also contribute to abnormal sleep regulation [60]. These mechanisms may help to explain the occurrence of REM sleep disturbances and parasomnias reported in some patients. In addition, non-specific factors common to movement disorders may contribute to sleep impairment, including micro-arousals caused by nocturnal motor symptoms and the potential effects of medications used for tremor control [33,60].

The pathophysiology of subtle bradykinesia in ET is also complex and likely involves dysfunction across multiple interconnected neural networks [13]. Among these, the LC may represent a potential contributor. Through its widespread noradrenergic projections, the LC modulates cortical excitability, cerebellar output, and basal ganglia activity—systems that are all critical for normal movement execution [35,36,37]. Demonstrating an association between bradykinesia features and insomnia would have strengthened the hypothesis that Lewy body pathology within the LC contributes to the development of bradykinesia in ET. However, the absence of such an association in our cohort suggests that other neural substrates may play a more prominent role. Previous studies have proposed that subtle changes in dopaminergic transmission may contribute to ET bradykinesia [13,28,29]. Moreover, the cerebellum, which is widely considered a key structure in ET pathophysiology, may contribute not only to tremor generation but also to alterations in voluntary movement execution [7,13,30,61,62,63,64]. This hypothesis is supported by the established role of the cerebellum in regulating movement timing and velocity [65,66]. Furthermore, clinical and experimental studies have shown that cerebellar disorders—including degenerative diseases, tumors, and ischemic lesions—can be associated with movement slowness [13]. Finally, cortical mechanisms may contribute to ET bradykinesia [13]. Recent neurophysiological studies have demonstrated abnormalities in primary motor cortex excitability in these patients. Specifically, ET patients show reduced corticospinal excitability, impaired intracortical inhibition, and reduced long-term potentiation-like plasticity within the primary motor cortex. Importantly, these alterations have been shown to correlate with reduced movement velocity during repetitive finger-tapping tasks, further supporting the hypothesis that cortical dysfunction may contribute to subtle bradykinesia in ET [31].

As already shown in our previous study on these participants in the context of a larger sample [5], which this work represents a further in-depth analysis of, we observed a significant association between insomnia and clinical tremor severity, with a rank-biserial correlation indicating a large effect size. In ET, insomnia may be more closely linked to the functional severity of tremor rather than to subtle bradykinesia. This supports a model in which sleep disturbances exacerbate the overall disability and patient-perceived burden of tremor [33]. The relationship between sleep disturbances and ET is complex and likely bidirectional. On one hand, sleep abnormalities may contribute to the clinical expression of ET by modulating central nervous system excitability, emotional regulation, and motor control [5,59,67,68,69]. On the other hand, sleep abnormalities may arise as a consequence of tremor severity and associated symptoms. Patients with more severe tremor often experience greater disability, social embarrassment, and performance anxiety, all of which can negatively affect sleep initiation and maintenance [5,20,70]. In addition, sleep disturbances may be influenced by pharmacological treatments commonly used in ET patients, including anxiolytics, beta-blockers, sedatives or antidepressants, which may affect both sleep and daytime functioning [71,72]. Finally, comorbid sleep disorders, such as obstructive sleep apnea, restless legs syndrome, and REM sleep behavior disorder, should be considered when interpreting our findings, given their potential impact on sleep architecture. From a clinical perspective, these findings highlight the importance of systematically assessing sleep quality in ET patients, particularly in those with more severe tremor, where sleep disturbances may be considered both a comorbidity to be screened for and a marker of disease burden. Notably, in contrast to the clinical measures, we did not observe correlations between insomnia severity and kinematic tremor parameters. This discrepancy may reflect differences between clinical rating scales and instrumental measurements. Clinical assessments capture not only objective motor impairment but also the functional consequences of tremor in daily activities, whereas kinematic analyses evaluate tremor characteristics in controlled experimental conditions and specific postures, that may not fully reflect real-life functional impairment. A further confirmatory finding is the lower MoCA scores observed in patients with insomnia. Recent evidence suggests that sleep disturbances in ET may serve as potential predictors of cognitive impairment [73]. This association may reflect shared underlying mechanisms, though other factors likely contribute. For instance, age-related changes in sleep–wake regulation, including alterations in melatonin secretion and circadian rhythms, may influence both sleep quality and cognitive performance. In addition, structural brain changes related to aging or emerging neurodegeneration may underlie the relationship between sleep disturbances and executive dysfunction. In this context, sleep abnormalities in ET may be interpreted as part of a broader potentially neurodegenerative framework [74], in which dynamic interactions between hemispheres and cortical–subcortical networks contribute to motor and non-motor dysfunctions [13,62,75,76,77].

Our study has several limitations. First, the sample size was relatively modest, which may have limited statistical power to detect subtle associations, and may have increased the possibility of a type II error. In addition, although the number of predictors was limited, the sample size may still have constrained the stability of the regression estimates, and a potential risk of overfitting cannot be entirely excluded. Second, the clinic-based and relatively older sample may limit the generalizability of our results to broader ET populations. The cross-sectional design prevents conclusions regarding causal relationships between motor features and sleep disturbances. Although a longitudinal approach would be more appropriate to address causality, the present study was specifically designed as an exploratory cross-sectional analysis aimed at identifying potential associations. Given the sample size, we did not perform Receiver Operating Characteristic curve analysis, as it would not yield robust estimates. Sleep disturbances were assessed using a self-reported questionnaire rather than objective measures such as polysomnography, and this may limit the ability to fully characterize sleep disturbances. However, the ISI demonstrated good internal consistency, with a Cronbach’s alpha value indicating adequate reliability [78]. Again, a single baseline sleep measure may not fully capture the dynamic evolution of sleep problems over time. Repeated assessments would be useful to determine whether sleep changes parallel disease progression or precede it. Regarding the kinematic analysis, the system employed in this study is an automated and standardized motion capture system, which minimizes operator-dependent variability and has demonstrated high reliability in previous reports. Moreover, the lack of direct LC imaging measures precludes the assessment of the relationship between LC structural alterations and clinical severity in our cohort. Finally, we did not assess environmental, cognitive, and social factors possibly contributing to cognitive and psychopathological manifestations in our patients [79]. Future studies involving larger cohorts, objective sleep recordings, and longitudinal follow-up will be important to better clarify the interaction between motor dysfunction and sleep disturbances in ET, and to determine whether sleep disturbances represent a trait marker, prodromal marker, or epiphenomenon in ET.

## 5. Conclusions

While subtle bradykinesia and insomnia are common and clinically significant symptoms in ET, they appear to be at least partially independent and likely driven by distinct underlying mechanisms involving broad neural networks. Our data indicate that poor sleep quality is specifically associated with greater clinically assessed tremor severity, carrying important clinical implications and providing further insight into the clinical heterogeneity of ET. Larger studies integrating kinematic assessments, neuroimaging, and longitudinal designs are needed to further clarify the relationship between motor and non-motor symptoms in ET.

## Figures and Tables

**Table 1:** Continuous clinical and kinematic variables in patients with essential tremor (ET) stratified according to the presence of bradykinesia. Patients were split into two subgroups: ET non-slow (ET-ns) and ET slow (ET-s) based on kinematic movement velocity values according to a median split procedure. TETRAS: The Essential Tremor Rating Assessment Scale, including the performance (TETRAS_P_) and the activity of daily living subscales (TETRAS_ADL_). UL: upper limbs. MDS-UPDRS Part III: Movement Disorder Society Unified Parkinson’s Disease Rating Scale, Part III. SARA: scale for the assessment and rating of ataxia. Insomnia Severity Index (ISI). PSS: Perceived Stress Scale. MoCA: Montreal Cognitive Assessment. HADS: Hospital Anxiety and Depression Scale. QoL: quality of life. HR-QoL: health-related quality of life. P1: posture 1. P2: posture 2. Tremor frequency is expressed in Hz, tremor amplitude in GRMS^2. Values are presented as mean ± standard deviation. *p* values from the Mann–Whitney U test. Significant values after correction for multiple comparisons are in bold.

	ET-ns	ET-s	*p*-Value	Rank-Biserial Correlation
*Clinical variables*				
Age	60.2 ± 15.5	73.4 ± 10.4	0.006	−0.595
Age at onset	41.7 ± 21.9	52.9 ± 18.7	0.221	0.267
TETRAS_TOT_	36.2 ± 15.6	36.4 ± 17.3	0.861	−0.038
TETRAS_ADL_	14.0 ± 8.32	13.7 ± 8.48	0.878	0.033
TETRAS_P_	22.2 ± 8.35	22.7 ± 11.0	0.913	−0.024
Action UL severity	7.70 ± 2.53	5.54 ± 3.71	0.046	0.433
Rest UL severity	1.20 ± 1.41	1.79 ± 1.95	0.375	−0.195
Body parts affected	2.40 ± 1.18	2.36 ± 1.39	0.786	0.057
MDS-UPDRS Part III	6.47 ± 4.10	15.4 ± 8.39	**0.001**	**−0.709**
SARA	2.43 ± 2.21	1.64 ± 1.54	0.343	0.205
ISI	7.00 ± 4.38	7.00 ± 6.23	0.677	0.090
PSS	15.3 ± 5.87	14.2 ± 5.66	1.000	0.000
MoCA	24.8 ± 2.57	26.9 ± 1.27	0.013	−0.533
HADS anxiety	6.07 ± 1.98	6.00 ± 4.39	0.311	0.000
HADS depression	5.40 ± 3.68	4.79 ± 3.66	0.742	0.071
QoL	74.0 ± 15.7	77.5 ± 20.1	0.241	−0.252
HR-QoL	54.3 ± 27.1	72.9 ± 20.5	0.047	−0.428
*Tremor kinematics*				
Rest tremor frequency	6.33 ± 0.73	5.48 ± 1.07	0.034	0.614
Rest tremor amplitude	0.02 ± 0.01	0.05 ± 0.07	0.283	0.314
Postural tremor frequency (P1)	5.34 ± 1.27	5.04 ± 0.49	0.386	0.250
Postural tremor amplitude (P1)	0.06 ± 0.05	0.08 ± 0.04	0.124	−0.444
Postural tremor frequency (P2)	6.28 ± 1.64	5.37 ± 1.31	0.123	0.343
Postural tremor amplitude (P2)	0.14 ± 0.15	0.43 ± 0.54	0.174	−0.302
Kinetic tremor	1.37 ± 1.23	1.08 ± 0.54	0.432	−0.171

**Table 2:** Categorical clinical variables in patients with essential tremor (ET) stratified according to the presence of bradykinesia. Patients were split into two subgroups: ET non-slow (ET-ns) and ET slow (ET-s) based on kinematic movement velocity values according to a median split procedure. F: female; FH: familiar history; Y: yes; LL: lower limbs; ISI: Insomnia Severity Index. Percentages are indicated in parentheses. *p* from Fisher’s exact test.

	ET-ns	ET-s	*p*-Value	φ Value
*Clinical variables*				
Sex	8 F (55.3%)	7 F (50.0%)	1.000	0.033
FH	8 Y (53.3%)	8 Y (57.1%)	1.000	0.038
Onset distribution	2 head (6.7%)	2 head (14.3%)	0.598	0.125
Head tremor	6 Y (40.0%)	5 Y (35.7%)	1.000	0.044
Face tremor	2 Y (13.3%)	3 Y (21.4%)	0.651	0.107
Voice tremor	7 Y (46.7%)	6 Y (42.49%)	1.000	0.038
LL tremor	6 Y (40.0%)	4 Y (28.6%)	0.700	0.120
Insomnia (ISI ≥8)	6 Y (40.0%)	5 Y (35.7%)	0.812	0.044
*Soft signs*				
Rest tremor	10 Y (66.7%)	9 Y (64.3%)	1.000	0.025
Subtle bradykinesia	4 Y (26.7%)	6 Y (42.9%)	0.450	0.170
Questionable dystonia	0 Y (0.0%)	1 Y (7.1%)	0.292	0.196
Impaired tandem gate	6 Y (40.0%)	4 Y (28.6%)	0.518	0.120
Cognitive disturbances	9 Y (60.0%)	2 Y (14.3%)	0.011	0.471
Vivid dreams	4 Y (26.7%)	2 Y (14.3%)	0.510	0.358

**Table 3:** Continuous clinical and kinematic variables in patients with essential tremor (ET) stratified according to the Insomnia Severity Index (ISI). Patients are divided into two subgroups: those without insomnia (ISI < 8) and those with insomnia (ISI ≥ 8). TETRAS: The Essential Tremor Rating Assessment Scale, including the performance (TETRAS_P_) and the activity of daily living subscales (TETRAS_ADL_). UL: upper limbs. MDS-UPDRS Part III: Movement Disorder Society Unified Parkinson’s Disease Rating Scale, Part III. SARA: scale for the assessment and rating of ataxia. PSS: Perceived Stress Scale. MoCA: Montreal Cognitive Assessment. HADS: Hospital Anxiety and Depression Scale. QoL: quality of life, HR-QoL: health-related quality of life. N mov: number of finger-tapping movements. CV: coefficient of variation. P1: posture 1. P2: posture 2. Movement amplitude in degrees, movement velocity is expressed in degrees/sec. Velocity and amplitude decrement are expressed in (degrees/sec)/n mov and (degrees/n mov), respectively. Tremor frequency is expressed in Hz, tremor amplitude in GRMS^2. Values are presented as mean ± standard deviation. *p* values from the Mann–Whitney U test. Significant values after correction for multiple comparisons are in bold.

	ET-ni	ET-i	*p*-Value	Rank-Biserial Correlation
*Clinical variables*				
Age	69.6 ± 10.3	61.5 ± 19.5	0.154	−0.166
Age at onset	49.3 ± 21.4	43.5 ± 20.4	0.482	−0.207
TETRAS_TOT_	29.4 ± 14.8	47.6 ± 11.6	**0.002**	**0.687**
TETRAS_ADL_	10.7 ± 7.33	19.0 ± 7.22	**0.006**	**0.606**
TETRAS_P_	18.6 ± 8.44	28.6 ± 8.11	**0.004**	**0.611**
Action UL severity	5.50 ± 3.00	8.55 ± 2.94	0.013	0.556
Rest UL severity	0.972 ± 1.34	2.32 ± 1.91	0.035	0.484
Body parts affected	2.22 ± 1.17	2.64 ± 1.43	0.402	0.161
MDS-UPDRS Part III	10.20 ± 6.80	11.6 ± 9.60	0.646	0.020
SARA	2.06 ± 2.01	2.05 ± 1.86	0.989	0.085
ISI	3.56 ± 2.36	12.6 ± 3.38	**<0.001**	**1.000**
PSS	16.7 ± 4.33	11.6 ± 6.45	0.018	−0.555
MoCA	26.6 ± 1.92	24.6 ± 2.42	0.025	−0.454
HADS anxiety	6.33 ± 2.89	5.55 ± 3.98	0.542	−0.181
HADS depression	4.83 ± 2.96	5.55 ± 4.63	0.616	0.005
QoL	77.2 ± 15.7	73.2 ± 21.1	0.561	−0.080
HR-QoL	63.6 ± 28.8	62.7 ± 20.4	0.930	−0.111
*Finger-tapping kinematics*				
N mov	40.9 ± 10.3	42.2 ± 10.3	0.739	0.156
Movement velocity	993 ± 270	1065 ± 266	0.489	0.141
Movement amplitude	47.1 ± 12.4	49.2 ± 8.15	0.653	0.101
CV	0.105 ± 0.0406	0.0883 ± 0.0429	0.616	−0.333
Amplitude decrement	−0.102 ± 0.255	−0.199 ± 0.183	0.282	−0.343
Velocity decrement	−4.61 ± 2.92	−7.82 ± 5.03	0.021	−0.515
*Tremor kinematics*				
Rest tremor frequency	6.10 ± 1.03	5.58 ± 1.00	0.306	−0.277
Rest tremor amplitude	0.06 ± 0.08	0.06 ± 0.08	0.213	−0.083
Postural tremor frequency (P1)	5.17 ± 1.02	5.17 ± 1.02	0.973	−0.027
Postural tremor amplitude (P1)	0.09 ± 0.06	0.09 ± 0.06	0.168	−0.305
Postural tremor frequency (P2)	6.14 ± 1.54	6.14 ± 1.54	0.233	−0.304
Postural tremor amplitude (P2)	0.19 ± 0.26	0.19 ± 0.26	0.190	0.219
Kinetic tremor	1.33 ± 1.12	1.33 ± 1.12	0.459	0.247

**Table 4:** Categorial clinical variables in patients with essential tremor (ET) stratified according to the Insomnia Severity Index (ISI). Patients are divided into two subgroups: those without insomnia (ISI < 8) and those with insomnia (ISI ≥ 8). F: female; FH: family history; Y: yes; LL: lower libs. Percentages are indicated in parentheses. *p* from Fisher’s exact test.

	ET-ni	ET-i	*p*-Value	φ Value
*Clinical variables*				
Sex	10 F (55.6%)	5 F (36.4%)	0.450	0.186
FH	11 Y (61.1%)	5 Y (45.5%)	0.466	0.153
Onset distribution	2 head (11.1%)	1 head (9.1%)	1.000	0.032
Head tremor	6 Y (33.3%)	5 Y (45.5%)	0.696	0.121
Face tremor	2 Y (11.1%)	3 Y (27.3%)	0.339	0.208
Voice tremor	9 Y (50.0%)	4 Y (36.4%)	0.702	0.133
LL tremor	4 Y (22.2%)	6 Y (54.5%)	0.114	0.330
*Soft signs*				
Rest tremor	10 Y (55.6%)	9 Y (81.8%)	0.234	0.268
Subtle bradykinesia	5 Y (55.6%)	5 Y (45.5%)	0.432	0.180
Questionable dystonia	0 Y (0.0%)	1 Y (9.1%)	0.379	0.242
Impaired tandem gate	6 Y (33.3%)	4 Y (36.4%)	1.000	0.039
Cognitive disturbances	5 Y (27.8%)	6 Y (54.5%)	0.240	0.268
Vivid dreams	4 Y (22.2%)	2 Y (18.2)	1.000	0.048
*Kinematic analysis*				
Subtle bradykinesia (movement velocity < median value)	9 Y (64.3%)	5 Y (35.7%)	1.000	0.044

**Table 5:** Correlation analysis between the Insomnia Severity Index (ISI) and clinical as well as kinematic variables in patients with essential tremor (ET). TETRAS: The Essential Tremor Rating Assessment Scale, including the performance (TETRAS_P_) and the activity of daily living subscales (TETRAS_ADL_). UL: upper limbs. MDS-UPDRS Part III: Movement Disorder Society Unified Parkinson’s Disease Rating Scale, Part III. SARA: scale for the assessment and rating of ataxia. PSS: Perceived Stress Scale. MoCA: Montreal Cognitive Assessment. HADS: Hospital Anxiety and Depression Scale. QoL: quality of life, HR-QoL: health-related quality of life. N mov: number of finger-tapping movements. CV: coefficient of variation. P1: posture 1. P2: posture 2. *p*-values are from Spearman correlation and have been corrected for multiple comparisons using the False Discovery Rate (FDR). Significant vales are in bold.

Variable	Spearman ρ	*p*-Value
*Clinical variables*		
Age	0.035	0.858
Age at onset	−0.002	0.991
TETRAS_TOT_	**0.581**	**<0.001**
TETRAS_ADL_	**0.525**	**0.003**
TETRAS_P_	**0.538**	**0.003**
Action UL Severity	**0.535**	**0.003**
Rest UL Severity	0.328	0.083
Body parts affected	0.059	0.762
MDS-UPDRS III	0.145	0.453
SARA	0.156	0.418
PSS	−0.323	
MoCA	−0.316	0.095
HADS anxiety	−0.061	0.755
HADS depression	0.093	0.630
HADS total	0.004	0.983
Hr-QoL	−0.201	0.296
QoL	−0.178	0.354
*Finger-tapping kinematics*		
N mov	0.059	0.762
Movement velocity	0.084	0.665
Movement amplitude	0.098	0.612
CV	−0.302	0.112
Amplitude Decrement	−0.251	0.190
Velocity Decrement	0.424	0.022
*Tremor kinematics*		
Rest tremor frequency	−0.371	0.143
Rest tremor amplitude	−0.323	0.205
Postural tremor frequency (P1)	**−** **0.420**	**0.026**
Postural tremor amplitude (P1)	**0.304**	**0.136**
Postural tremor frequency (P2)	**−0.430**	**0.022**
Postural tremor amplitude (P2)	0.127	0.519
Kinetic tremor	0.168	0.385

## Data Availability

The data supporting the findings of this study are not publicly available due to privacy restrictions but are available from the corresponding author upon reasonable request.

## Supplementary Materials

The following supporting information can be downloaded at: https://www.mdpi.com/article/10.3390/brainsci16050504/s1, Table S1: Comorbidities in patients with essential tremor (ET).

## Abbreviations

The following abbreviations are used in this manuscript:

ADL	activity of daily living
CI	curvature index
CV	coefficient of variation
ET	essential tremor
ET-i	essential tremor with insomnia
ET-ni	essential tremor without insomnia
ET-ns	essential tremor non slow
ET-s	essential tremor slow
FDR	false discovery rate
HADS	hospital anxiety and depression scale
HR-QoL	health related quality of life
ISI	insomnia severity index
LC	locus coeruleus
MDS-UPDRS	Movement Disorder Society-sponsored revision of the unified Parkinson’s disease rating scale
MoCA	Montreal cognitive assessment
PD	Parkinson’s disease
PSS	perceived stress scale
QoL	quality of life
RMS	root mean square
SARA	scale for the assessment and rating of ataxia
SD	standard deviation
TETRAS	the essential tremor rating assessment scale
TETRAS_ADL_	TETRAS activities of daily living subscale
TETRAS_P_	TETRAS performance subscale
TETRAS_TOT_	TETRAS total score
