# Supplementary material for: Is There a Relationship Between Movement and Sleep Disturbances in Essential Tremor?
Source: Brain Sci. 2026 May 7;16(5):504. doi: 10.3390/brainsci16050504 (PMC13204725; doi:10.3390/brainsci16050504)
Supplement: Supplementary file 1 [file brainsci-16-00504-s001.zip › brainsci-4263058-supplementary.pdf]

SUPPLEMENTARY MATERIALS

Pt	Comorbidities
1	-
2	Hypothyroidism, osteoporosis
3	Arterial hypertension
4	Arterial hypertension
5	Cerebral amyloid angiopathy, cerebral hemorrhage, secondary epilepsy
6	Arterial hypertension, type 2 diabetes
7	Arterial hypertension, dyslipidemia
8	-
9	Benign prostatic hyperplasia
10	-
11	Arterial hypertension, dyslipidemia
12	Arterial hypertension
13	-
14	-
15	Dyslipidemia, non-functioning pituitary adenoma, polycystic ovary syndrome
16	Polymyalgia rheumatica, Type 2 diabetes
17	Dyslipidemia, Hashimoto's thyroiditis, rheumatoid arthritis
18	Arterial hypertension, benign prostatic hyperplasia, dilated cardiomyopathy, dyslipidemia
19	-
20	-
21	-
22	-
23	-
24	-
25	Hemophilia A, diverticulosis

26	Arterial hypertension
27	Dyslipidemia
28	Benign prostatic hyperplasia, multinodular goiter
29	Arterial hypertension, dyslipidemia

Supplementary Table 1. Comorbidities in patients with essential tremor (ET).
